# Cyclopeptide 161 protects human dermal fibroblasts against UV-induced damage via the MEK1/2–ERK1/2 signaling pathway

**DOI:** 10.3389/fphys.2026.1912562

**Published:** 2026-09-09

**Authors:** Yue Xiao, Xingge Huang, Xiao Zhang, Guanlin Li, Bing Tian, Jing Wang, Xiao Feng, Zhe Feng, Binqiang Wang, Hu Huang, Hong Xu

**Affiliations:** 1 Ministry of Education (MOE), Key Laboratory of Biosystems Homeostasis and Protection, Institute of Biophysics, College of Life Science, Zhejiang University, Hangzhou, China; 2 Proya Cosmetics Co., Ltd, Hangzhou, China

**Keywords:** cyclopeptide 161, extracellular matrix, MAP2K1, Munc18a, proteomic analysis, UV irradiation

## Abstract

**Introduction:**

Cutaneous homeostasis is constantly perturbed by ultraviolet (UV) irradiation, which triggers extracellular matrix (ECM) structural derangement, fibroblast senescence, and the gradual deterioration of dermal integrity. CP161, a synthetic cyclic hexapeptide, has been documented to upregulate the transcription of collagen genes. Nevertheless, its broader cutaneous bioactivities as well as the precise molecular mechanisms underlying its skin-regulating effects remain largely unelucidated.

**Methods:**

We first characterized the skin-protective potential of CP161 in primary human dermal fibroblasts (HDFs) exposed to ultraviolet radiation, relying on RT-qPCR, Western blot analysis, SA-β-gal staining and intracellular reactive oxygen species quantification for phenotypic validation. We utilized label-free quantitative proteomics to map core biological events and signaling networks altered following CP161 treatment, and adopted CETSA, BLI, molecular docking together with molecular dynamics (MD) simulations to verify whether CP161 can physically bind MAP2K1. We extended our investigation to cultured cholinergic neurons and PTZ-stimulated zebrafish larvae to further dissect CP161 biofunction; we quantified changes in acetylcholine (ACh) secretion and excessive neuromuscular activity in these two models, and similarly deployed CETSA, molecular docking and MD simulation assays to test for direct binding between CP161 and Munc18a.

**Results:**

In UVA-irradiated HDFs, CP161 restored *COL1A1*, *COL14A1*, and *COL16A1* expression, suppressed MMP1 and p21 upregulation, reduced SA-β-gal-positive cells, and lowered intracellular ROS accumulation. Proteomic profiling revealed coordinated remodeling of ECM organization. CP161 attenuated UVA-induced MEK1/2–ERK1/2 phosphorylation, and CETSA, BLI, molecular docking, and MD analyses collectively supported direct interaction between CP161 and MAP2K1. In cholinergic neurons, CP161 reduced evoked ACh release; in PTZ-challenged zebrafish larvae, CP161 dose-dependently attenuated neuromuscular hyperactivity, and convergent biophysical and computational evidence supported direct interaction between CP161 and Munc18a, indicating a complementary activity relevant to contraction-associated mechanical injury of the dermis.

**Conclusion:**

CP161 protects human dermal fibroblasts from UVA-induced damage through direct interaction with MAP2K1 and attenuation of the MEK1/2–ERK1/2 signaling cascade, thereby preserving ECM homeostasis. These findings establish a mechanistic framework for the skin-protective activity of CP161 and identify the MAP2K1–ERK1/2 axis as the principal pathway through which this cyclic hexapeptide maintains dermal homeostasis.

## 1 Introduction

Skin serves as the primary barrier separating an organism from its surrounding environment, yet its stable homeostatic state faces constant disruption from external harmful stimuli alongside endogenous mechanical tension (Jiao et al., 2024). Of all these external stressors, ultraviolet (UV) radiation stands out as the dominant trigger responsible for skin photoaging. It accounts for most clinically noticeable aging phenotypes on sun-exposed skin areas and creates a considerable global public health and cosmetic concern (Shin et al., 2019, 2023). Histologically, damaged skin is characterized by progressive disorganization of the dermal extracellular matrix (ECM), fragmentation of fibrillar collagens, elastotic degeneration, accumulation of senescent fibroblasts, and progressive loss of dermal mechanical integrity (Csekes and Rackova, 2021; Quan, 2023). Dermal fibroblasts sit at the core of this pathological progression. These cells synthesize the bulk of ECM constituents, sense mechanical and oxidative signals inside dermal tissue, and their gradual functional decay ultimately drives most visible signs of skin senescence (Tigges et al., 2014; Fitsiou et al., 2021).

At the molecular level, UVA irradiation initiates a cascade of intracellular events that converge on the mitogen-activated protein kinase (MAPK) signaling network. UVA generates reactive oxygen species (ROS) through both direct photochemical reactions with cellular chromophores and indirect activation of mitochondrial and NADPH oxidase–dependent sources (Cadet and Wagner, 2013; Gu et al., 2020; Godar, 2021). The resulting oxidative burst produces lipid peroxidation, oxidative DNA lesions, and oxidation-induced inactivation of protein tyrosine phosphatases, which together promote sustained ligand-independent activation of upstream receptor tyrosine kinases (Cavinato and Jansen-Durr, 2017; Sample and He, 2018). Activation of these receptors triggers the canonical MEK–ERK cascade, in which the dual-specificity kinase MAP2K1 (MEK1) and its paralog MAP2K2 (MEK2) phosphorylate ERK1/2 on conserved threonine and tyrosine residues (Roskoski, 2019; Lavoie et al., 2020). The MEK1/2–ERK1/2 axis couples upstream UVA-triggered signals to a transcriptional program that promotes matrix metalloproteinase 1 (MMP1) induction, upregulates the cyclin-dependent kinase inhibitor p21, and suppresses fibrillar collagen genes including *COL1A1*, *COL14A1*, and *COL16A1* (Caunt et al., 2015; Quan and Fisher, 2015; Pittayapruek et al., 2016; Roskoski, 2019; Lavoie et al., 2020). Sustained activation of this cascade drives collagen fragmentation, fibroblast cell-cycle arrest, and disorganization of the dermal ECM (Wang and Dreesen, 2018; Shin et al., 2019; Csekes and Rackova, 2021; Pilkington et al., 2021).

Within this cascade, MAP2K1 sits at a rate-limiting node, and for that reason it has come to be viewed as a therapeutically appealing entry point for modulating MAPK-driven pathology (Roskoski, 2012; Kun et al., 2021). Its constitutive activation is, of course, a well-characterized oncogenic driver in cutaneous melanoma and also a principal contributor to RASopathies like cardio-facio-cutaneous syndrome—which really underscores just how central this kinase is to cutaneous biology (Tajan et al., 2018; Hebron et al., 2022). Pharmacological inhibitors developed for oncology—trametinib, cobimetinib, binimetinib—have certainly validated MAP2K1 as druggable, yet their dermatologic toxicity profiles are substantial enough to rule out any use in cosmetic or general dermatology settings (Anforth et al., 2013; Caunt et al., 2015; Lacouture et al., 2021). Despite the well-established mechanistic role of the MAPK–ECM axis in photoaging, pharmacological strategies that selectively dampen this pathway in the skin without producing systemic toxicity remain limited (Rinnerthaler et al., 2015; Papaccio et al., 2022). A previous study reported that the plant-derived compound VB1 binds MAPK1/ERK2 in UVA-irradiated fibroblasts (Wang et al., 2020), providing initial evidence that MAPK pathway components are druggable targets for anti-photoaging ingredients. However, whether other classes of anti-photoaging compounds, such as synthetic cyclic peptides, engage distinct nodes within this cascade remains unexplored.

Against this background, synthetic peptides have emerged as a particularly attractive class of skin-active molecules. Backbone cyclization restricts conformational freedom, lowers the entropic cost of binding, and frequently translates into improved affinity, selectivity, and resistance to proteolysis relative to linear peptides of identical sequence (Zorzi et al., 2017; Vinogradov et al., 2019; Buckton et al., 2021; Muttenthaler et al., 2021; Garcia Jimenez et al., 2023). These properties have made cyclic peptides effective at engaging shallow protein surfaces and protein–protein interfaces that small molecules struggle to address (Wang and Craik, 2018; Dougherty et al., 2019; Sohrabi et al., 2020; Ji et al., 2024). Within this broad chemical space, synthetic cyclic hexapeptides occupy a particularly useful middle ground: they are large enough to make multiple specific contacts with a protein target, yet small enough to remain synthetically tractable and amenable to systematic structural analysis (Rhodes and Pei, 2017; Goto and Suga, 2021; Bhardwaj et al., 2022; Merz et al., 2024). The chemical space occupied by cyclic peptides bridges the gap between small-molecule and biologic therapeutics and has been progressively exploited in the design of next-generation skin-active compounds (Apostolopoulos et al., 2021; Buckton et al., 2021).

Cyclopeptide 161 (CP161) is a synthetic cyclic hexapeptide that has been reported, in a patent disclosure (CN114634554A), to promote collagen-related gene expression. Although this phenotypic activity is consistent with a potential role in mitigating dermal ECM loss, the mechanism of CP161 and the molecular target through which it operates have not been defined. The absence of a mechanistic framework has limited rational optimization of this peptide and has precluded a clear understanding of how its phenotypic activity relates to the established drivers of dermal senescence.

In addition to its reported activity, preliminary observations from our laboratory indicated that CP161 also modulates evoked neurotransmitter release in cholinergic neurons. Evoked exocytosis at presynaptic terminals is executed by the SNARE complex, whose stepwise zippering drives vesicle–membrane fusion (Han et al., 2017; Brunger et al., 2018; Rizo, 2022; Jahn et al., 2024). SNARE assembly is licensed by Munc18a (also known as STXBP1), a Sec1/Munc18 family protein that holds syntaxin-1 in a closed conformation, chaperones it to active zones, and templates productive trans-SNARE assembly (Misura et al., 2000; Baker and Hughson, 2016; Lai et al., 2017; Stepien et al., 2022). Genetic ablation of Munc18a abolishes evoked neurotransmitter release, placing it at an obligatory checkpoint upstream of vesicle fusion (Verhage et al., 2000; Sitarska et al., 2017). At cholinergic neuromuscular junctions, attenuation of Munc18a-dependent SNARE assembly reduces acetylcholine (ACh) discharge and downstream muscle contraction (Slater, 2017; Chen et al., 2020; Abramov et al., 2021). Repeated contraction of facial muscles, in turn, imposes recurrent mechanical strain on the overlying dermis and contributes to the dermal senescence (Cotofana et al., 2016; de Maio, 2018; Suwanchinda et al., 2018). A peptide that directly binds Munc18a and dampens its function would therefore be expected to soften neuromuscular drive on the dermis, offering one molecular route by which the structural integrity of skin could be preserved.

The present study was designed to define the molecular basis of the skin-protective activity of CP161 in UVA-irradiated human dermal fibroblasts. Using a combination of phenotypic characterization, label-free quantitative proteomics, and orthogonal target-engagement assays including cellular thermal shift assay (CETSA), biolayer interferometry (BLI), molecular docking, and molecular dynamics (MD) simulation, we identify MAP2K1 as a direct binding partner of CP161 and demonstrate that engagement of this kinase attenuates UVA-induced MEK1/2–ERK1/2 phosphorylation, restores fibrillar collagen gene expression, suppresses MMP1 and p21 induction, and reduces intracellular ROS. As an extended characterization of the broader bioactivity of this peptide, we further show that CP161 directly engages Munc18a and attenuates evoked ACh release, an activity that is recapitulated in a zebrafish neuromuscular hyperactivity model. Together, these findings establish a mechanistic framework for the skin-protective activity of CP161 centered on the MAP2K1–ERK1/2–ECM axis, and reveal an additional dimension of its bioactivity in cholinergic neurotransmission.

## 2 Materials and methods

### 2.1 Chemical reagents and antibodies

His-MAP2K1 protein and His-Munc18a protein were synthesized by Qingke Biotechnology (China). HIS1K biosensors was purchased from Sartorius (Germany). Primary antibodies against p21 (ab109520, Abcam, USA), MMP1 (10371-2-AP, proteintech, China), MEK1/2 (ab278723, Abcam, USA), p-MEK1/2 (ab194754, Abcam, USA), ERK1/2 (ab184699, Abcam, USA; T40071, Abmart, China) and p-ERK1/2 (ab201015, Abcam, USA; TA1015, Abmart, China) were used, whereas β-actin (ab8226, Abcam, USA) was used separately as internal controls. Primary antibody targeting β-actin was applied at a dilution of 1:2000, while all other primary antibodies were utilized at 1:1000. Proteins were detected by incubation with secondary antibodies (1:5,000): Goat anti-Rabbit IgG (ab97051, Abcam, USA) or Goat anti-Mouse IgG (ab97040, Abcam, USA).

### 2.2 Cell culture and treatment

Cells were cultured in low-glucose Dulbecco’s Modified Eagle’s Medium (DMEM; Gibco, Waltham, USA) supplemented with 10% fetal bovine serum (FBS; Gibco, Waltham, USA) and 1% penicillin–streptomycin solution (Gibco, Waltham, USA), and maintained at 37 °C in a humidified incubator with 5% CO_2_ overnight.

For the UVA-treated model, HDFs were pretreated with CP161 for 4 h before UVA irradiation. Prior to irradiation, the culture medium was replaced with PBS, and the cells were exposed to UVA at a dose of 25 J/cm² for 55 min. After irradiation, PBS was removed and replaced with CP161-containing complete medium, and the cells were incubated overnight. For the second irradiation cycle, the culture medium was again replaced with PBS before UVA exposure, followed by irradiation at 25 J/cm² for 55 min. After the second irradiation, PBS was replaced with CP161-containing complete medium, and the cells were incubated overnight. This two-cycle UVA exposure protocol was used to establish the UVA-induced injury HDFs.

### 2.3 Cell viability assay

The cellular viability was evaluated via the CCK-8 detection kit obtained from Beyotime Biotechnology (China). HDFs were inoculated into 96-well culture plates with a seeding density of 8 × 10³ cells per well, followed by an overnight incubation period to facilitate cell attachment. Next, these cells were exposed to CP161(Purity≥ 99.0%) at a series of gradient concentrations and cultured under a constant temperature of 37 °C for either 24 hours or 48 hours. Upon the completion of drug intervention, 10 microliters of CCK-8 working solution were dispensed into every single well, and the culture plates were kept incubating at 37 °C for another 1 to 2 hours. A microplate spectrophotometer manufactured by BioTek Instruments (USA) was applied to record optical density values at the wavelength of 450 nm.

### 2.4 Western blot

HDFs were placed on ice and subjected to lysis treatment for 30 minutes with radioimmunoprecipitation assay lysis solution supplemented with 1% phenylmethylsulfonyl fluoride (purchased from Beyotime, China). The resulting mixture underwent centrifugation at 12,000 rpm under 4 °C for 15 min, and the collected supernatant was adopted to quantify total protein concentration with the Enhanced BCA Protein Quantification Kit (Thermo Fisher Scientific, USA). Extracted total proteins were fractionated via electrophoresis on 10% sodium dodecyl sulfate-polyacrylamide gels, followed by electrotransfer onto polyvinylidene difluoride membranes supplied by Merck Millipore, Germany. A fixed amount of 25 μg total protein was loaded per well to ensure the chemiluminescence signals of proteins fell within the linear detection range. To avoid potential cross-reactivity or incomplete stripping, all proteins were incubated with primary antibodies on separate PVDF membranes without membrane stripping and re-probing. For blocking non-specific binding sites, the prepared membranes were immersed in TBST solution containing 5% skimmed milk for a 1-hour incubation period. Afterwards, these membranes were incubated with corresponding primary antibodies at 4 °C throughout an entire night. Subsequent incubation with matched secondary antibodies enabled immunological recognition of target protein molecules. SuperSignal West Pico Plus Chemiluminescent Substrate (Thermo Fisher Scientific, USA) was utilized to display specific protein bands, and the relative expression quantity of each protein was quantified and processed for statistical analysis relying on ImageJ analytical software, based on the original uncropped membrane images (provided in **Supplementary Figures S5–S8**).

### 2.5 RNA extraction and quantitative real-time PCR

Total cellular RNA was isolated from samples with the TRIzol extraction reagent. Reverse transcription reactions were carried out with PrimeScript™ 1st Strand cDNA Synthesis Kit (Takara, Japan) to synthesize complementary DNA templates. Quantitative real-time PCR amplification was implemented relying on TB Green Premix Ex Taq™ (Takara, Japan), with amplification signals detected on the QuantStudio 3 thermal cycler produced by Applied Biosystems (Thermo Fisher Scientific, USA). To calculate relative transcript abundance of the tested genes, all measured data were normalized against the internal reference gene GAPDH transcript levels. The comparative 2−ΔΔCT algorithm was adopted for subsequent statistical calculation of gene expression data. Detailed sequences for all designed amplification primers can be found in **Supplementary Table 1**. Primer efficiency standard curves can be found in **Supplementary Figure 4**.

### 2.6 Cell thermal shift assay and proteome-wide target identification

CETSA was performed using cell lysates prepared by ice-cold sonication. Protein concentrations were determined by BCA assay and normalized before aliquoting into eight equal portions (100 μg each). Samples were separately heated at 37, 42, 47, 52, 57, 62, 67, and 72 °C for 3 min, then cooled and centrifuged at 20,000 × g for 20 min at 4 °C. Soluble proteins were collected for reduction, alkylation, overnight trypsin digestion, desalting with MonoSpin C18, and LC-MS/MS analysis. MS acquisition was performed on a timsTOF Pro 2 system in DDA mode, raw data were processed using MaxQuant. Thermal stability curves were fitted for all quantified proteins using the TPP R package with non-linear regression, where each protein’s relative abundance at each temperature (normalized to its abundance at 37 °C) was fitted to a sigmoidal melting curve. The melting temperature (Tm, defined as the temperature at which 50% of the protein is denatured) was calculated for each protein in both CP161-treated and vehicle control groups, and the Tm shift (ΔTm = Tm-CP161 – Tm-control) was used as the primary metric for direct ligand–protein engagement. Proteins exhibiting ΔTm ≥ 5 °C with curve-fitting R² ≥ 0.8 were considered candidate direct targets.

### 2.7 Reactive oxygen species measurement

ROS levels in HDFs were determined using a ROS Assay Kit (Beyotime, China) according to the manufacturer’s instructions. Following cell treatment, culture medium was removed and cells were washed once with 1 mL of HBSS. After aspiration, 500 μL of DCFH-DA probe (diluted in HBSS) was added to each well. Cells were incubated at 37 °C in a 5% CO_2_ atmosphere for 30 min. Subsequently, the probe solution was removed and cells were washed twice with HBSS to eliminate excess reagent. Cells were then trypsinized, collected by centrifugation, washed twice with HBSS, and resuspended in 300 μL of HBSS. Fluorescence intensity was immediately analyzed using a NovoCyte flow cytometer (Agilent Technologies, USA) with excitation at 488 nm. Cells were first gated on FSC-A vs SSC-A to exclude debris. Single cells were further identified using FSC-A vs FSC-H dot plot to eliminate cell doublets and aggregates. A minimum of 10,000 single-cell events per sample were acquired; The mean fluorescence intensity (MFI) of DCF (FITC channel) within single-cell population was quantified to evaluate intracellular ROS levels.

### 2.8 Biolayer interferometry

The binding affinity between cyclic peptide-161 and His-tagged MAP2K1 and Munc18a were evaluated using an Octet R2 system (Sartorius, Germany) with Anti-Penta-HIS (HIS1K) biosensors (Sartorius, Germany). PBST buffer (0.05% Tween-20) was used as the equilibration buffer, dilution buffer, and blank control to eliminate non-specific binding.

The His-MAP2K1 protein and His-Munc18a with a concentration of 1 mg/mL, was immobilized on the HIS1K biosensors: the biosensors were first equilibrated in PBST for 10 min, then immersed in the His-MAP2K1 protein solution for capture (300 s) until a stable signal plateau was achieved, followed by a 120 s rinse in PBST to remove unbound free protein. CP161 was serially diluted to final concentrations using PBST. The protein-immobilized biosensors were sequentially dipped into each CP161 solution for association (60 s) and then transferred to blank PBST for dissociation (60 s), with real-time interference pattern shifts recorded throughout the process. The group with only immobilized recombinant protein (without CP161) served as the negative control to correct for background signals and sensor drift. BLI kinetic curves were analyzed by steady-state equilibrium fitting using Octet Analysis Studio 13.0.52(Sartorius).

### 2.9 Senescence-associated β-galactosidase staining

SA-β-gal staining was conducted with the Lysosomal β-Galactosidase Staining Kit supplied by Beyotime (China), and all operational steps strictly followed the official protocol provided by the reagent manufacturer. In brief, cell culture supernatants were discarded first, followed by a single rinse step using HBSS buffer to wash adherent cells. One milliliter of fixing solution matching the β-gal staining system was added for cell immobilization, and samples remained at ambient temperature over a 15-minute fixation period. Upon finishing fixation treatment, residual fixative liquid was aspirated away, and cells received three repeated HBSS rinses, with each washing step lasting 3 minutes. Each culture well was supplemented with 1 mL prepared staining working liquid afterward, and the plates were placed under incubation conditions at 37 °C for an entire night. Parafilm wrapping was applied to seal the outer edge of the culture plate, which effectively avoided liquid solvent loss during long-term incubation. Once the incubation procedure ended, residual staining liquid was discarded, and cells underwent one final rinse using 1 mL HBSS solution. Micrographs were acquired with a Nikon ECLIPSE Ts2R bright-field light microscope equipped with a 10× phase-contrast objective lens, and the exposure time was set to 300 ms; afterward, positive cells were counted manually across a fixed number of randomly selected, non-overlapping fields per well by an observer blinded to treatment group.

### 2.10 Molecular docking and molecular dynamics simulation

The crystal structures of human specificity MAP2K1 (PDB ID: 4AN3) and human Munc18a (PDB ID: 3C98) were retrieved to serve as receptor models. Both proteins were prepared by performing structural optimization, adding hydrogen atoms, and assigning charges. The docking ligand, CP161, was prepared using AutoDockTools[43]. Molecular docking was conducted using AutoDock Vina [44]. For MAP2K1, the docking search space was centered on the inhibitor binding site (x = -1.23, y = 34.34, z = 9.19) with a radius of 15 Å. For Munc18a, the docking site was defined at the contact interface between domain 3a and the Syntaxin-1 H3 domain (x = 50.57, y = 71.40, z = 11.15) with a 15 Å radius.

The optimal docking conformation was subjected to molecular dynamics (MD) simulation using GROMACS 2019 on the MaxFlow platform (https://maxflow.ilabpower.com). The AMBER14SB and GAFF2 force fields were employed for standard and non-standard residues, respectively. The system was solvated with explicit water molecules using the TIP3P model, followed by the addition of sodium (Na^+^) and chloride (Cl^-^) ions for charge neutralization. The geometry and energy of the system were optimized using the steepest descent algorithm to reach a local energy minimum. Temperature equilibration was subsequently performed in the NVT ensemble at 298 K for 1 ns. Finally, a 75 ns production MD simulation was performed, using the V-rescale thermostat and the Parrinello-Rahman barostat for temperature and pressure coupling, respectively. MD trajectory analysis was performed using the MaxFlow platform. The statistical analysis of the Root Mean Square Deviation (RMSD) and the MM-PBSA binding free energy calculations were strictly performed on the equilibrated portion of the trajectories, after the complexes had reached thermodynamic equilibrium. Structural analysis and figure preparation were visualized using Open-Source PyMOL (Version 3.1.0). Two-dimensional (2D) protein-ligand interaction diagrams were generated using the PoseView tool via the Proteins.plus web server (https://proteins.plus/) to visualize the hydrogen bonding networks and hydrophobic interactions within the binding pockets.

### 2.11 Proteomic analysis

Proteomic profiling was conducted by Shanghai Meiji Bio-technology Co., Ltd. (Shanghai, China) using a DIA-based mass spectrometry platform. The study included three groups of samples: fibroblasts (BC), UVA-induced photoaged fibroblasts (NC), and UVA-induced photoaged fibroblasts treated with CP161, with three biological replicates analyzed for each group (n = 3). Briefly, cells were snap-frozen, homogenized in lysis buffer, and sonicated for protein extraction; the extracted proteins were quantified, trypsin-digested, and desalted. Equal amounts of digested peptides were reconstituted in MS loading buffer and separated using a Vanquish Neo nano-UHPLC system (Thermo Fisher Scientific) coupled with a uPAC High Throughput column (75 μm × 5.5 cm, Thermo, USA). Mobile phase A contained water with 2% acetonitrile and 0.1% formic acid, mobile phase B contained water with 80% acetonitrile and 0.1% formic acid, with a total elution duration of 8 min. Mass spectrometry detection was carried out on an Orbitrap Astral mass spectrometer (Thermo Fisher Scientific) in positive-ion DIA mode with electrospray voltage of 1.5 kV and MS1 scan range m/z 100–1700; raw data acquisition was controlled by Xcalibur 4.7 software.

Raw DIA spectra were processed with Spectronaut™ 19. Search settings: trypsin/P digestion, maximum 2 missed cleavages, peptide length 7–52 amino acids; fixed modification: carbamidomethyl (C); variable modifications: oxidation (M), acetyl (Protein N-term). Filter criteria: Protein FDR ≤ 0.01, Peptide FDR ≤ 0.01, Peptide Confidence ≥ 99%, XIC width ≤ 75 ppm. Protein quantification was calculated via the built-in MaxLFQ algorithm developed in MaxQuant.

### 2.12 DPPH radical scavenging assay

A fresh DPPH working solution (0.1 mg/mL) was prepared in 95% ethanol and maintained in the dark throughout the procedure. The assay was performed in 96-well plates with triplicate wells for each condition, including blank control, blank background, sample, and sample background groups. In each well, 100 μL of sample solution or solvent was combined with 100 μL of DPPH solution or 95% ethanol according to a preset layout. After gentle mixing, the mixtures were incubated at room temperature in the dark for 30 min, followed by absorbance measurement at 517 nm using a microplate reader.

The DPPH radical scavenging rate was calculated as follows:

Scavenging rate (%) = [1 − (T − T_0_)/(C − C_0_)] × 100

where T indicates the absorbance of the sample group, T_0_ the absorbance of the sample background, C the absorbance of the blank control, and C_0_ the absorbance of the blank background.

### 2.13 Zebrafish PTZ-induced locomotor activity assay

Five-day-post-fertilization (5 dpf) zebrafish larvae were used to establish a PTZ-induced hyperlocomotion model. Larvae were randomly assigned to 96-well plates (24 larvae per group) and co-exposed to 7.5 mM PTZ and the indicated samples. A blank control group and a PTZ-only group were included for comparison. Following 30 min of exposure, larval locomotor behavior was monitored for 20 min using a DanioVision behavior recording system (Noldus, Wageningen, The Netherlands). Movement tracking and quantitative analysis were performed using EthoVision XT infrared tracking software. The mobility of each larva was automatically calculated and used for subsequent statistical analysis.

### 2.14 Acetyl choline measurement assay

ACh release was measured in hippocampal neuronal cells using an ACh assay kit (Nanjing Jiancheng Bioengineering Institute, China) according to the manufacturer’s instructions. Cells were seeded in 6-well plates and cultured overnight until the confluence reached approximately 40–60%. The cells were then treated with the indicated samples at the specified concentrations and incubated for 24 h at 37 °C in a humidified incubator with 5% CO_2_. Each treatment was performed in triplicate, and untreated cells were included as the blank control. Following incubation, culture supernatants were collected and subjected to colorimetric determination of ACh content. Absorbance was measured using a microplate reader, and ACh levels were calculated relative to the control group. The inhibition rate was calculated using the following equation:

Inhibition rate (%) = [(Control − Sample)/Control] × 100.

### 2.15 Statistical analysis

All experiments were performed at least three times. Representative data were presented as means ± standard deviation (SD) and were analyzed using SPSS. For comparisons between two groups, Student’s t test was used. For comparisons among more than two groups, one-way analysis of variance (ANOVA) was used. *p < 0.05, **p < 0.01, ***p < 0.001 and ****p < 0.0001 were considered significant.

## 3 Results

### 3.1 CP161 alleviates UVA-induced senescence and oxidative stress responses in human dermal fibroblasts

**Figure 1:**
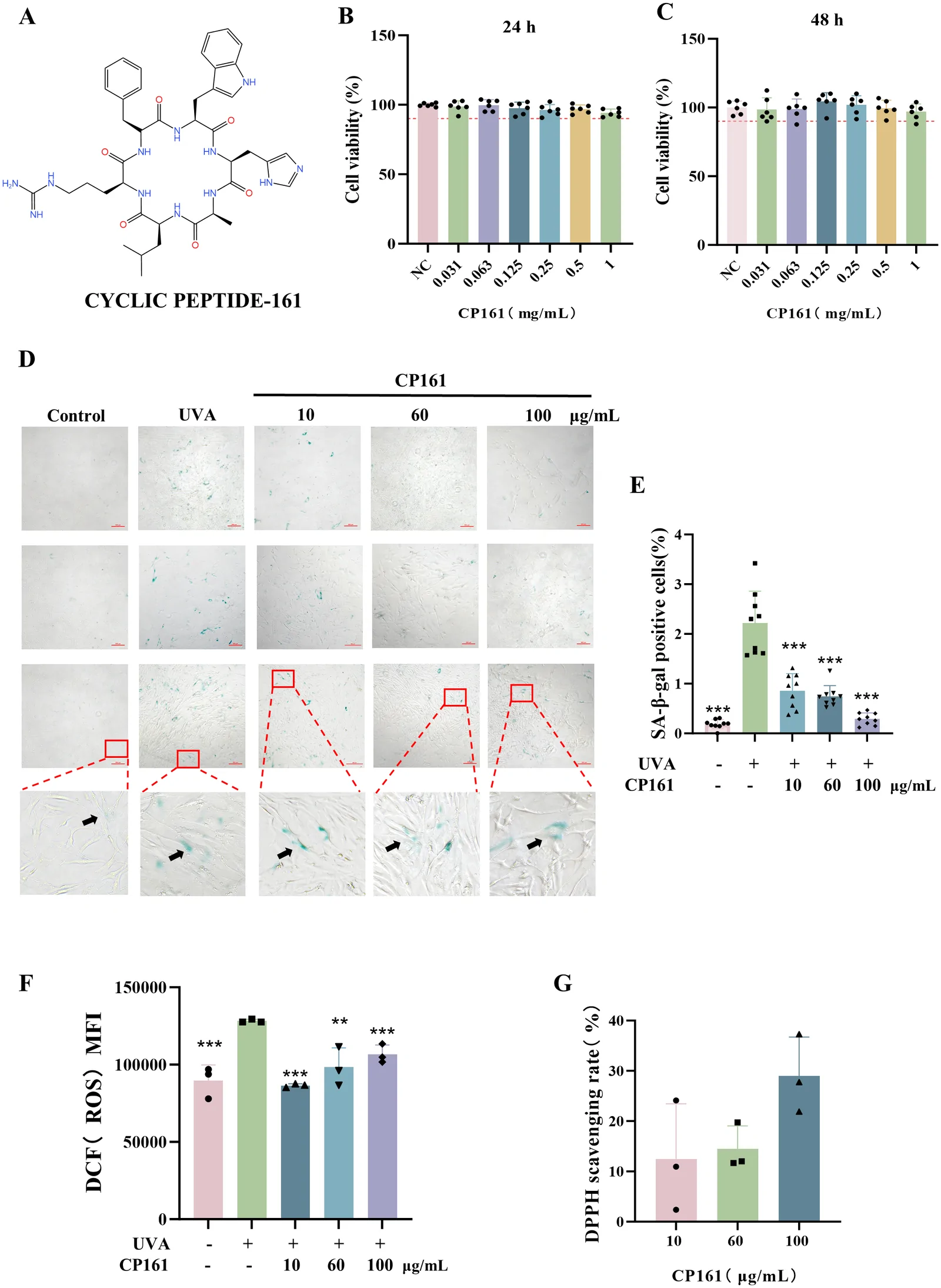
CP161 alleviates UVA-induced senescence and oxidative stress responses in HDFs. **(A)** Chemical structure of CP161. **(B, C)** Cytocompatibility of CP161 in HDFs determined by the CCK-8 assay. HDFs were treated with CP161 at 0.031, 0.063, 0.125, 0.25, 0.5, and 1 mg/mL for 24 h **(B)** and 48 h **(C)**, with untreated cells as the negative control (NC), n=6. **(D)** Representative micrographs of SA-β-gal staining in HDFs. Cells were exposed to UVA irradiation in the presence or absence of CP161 (10, 60, and 100 μg/mL), with untreated cells as the control. Scale bars = 500 μm. Magnified views of representative images are presented in the bottom row. Black arrows indicate representative SA-β-gal-positive cells. **(E)** Quantification of SA-β-gal-positive cells. ***P < 0.001 versus the UVA-only group. n=9. **(F)** ROS levels in HDFs measured by flow cytometry. Cells were exposed to UVA irradiation in the presence or absence of CP161 (10, 60, and 100 μg/mL). Mean fluorescence intensity (MFI) of DCF is shown. **P < 0.01 and ***P < 0.001 versus the UVA-only group, n=3. **(G)** DPPH radical scavenging activity of CP161 at 10, 60, and 100 μg/mL, expressed as the scavenging rate (%), n=3.

CP161 is a cyclic hexapeptide with the full amino acid sequence Cyclo (His-Ala-Leu-Arg-Phe-Trp-), the chemical structure of which is shown in **Figure 1A**. All amino acid monomers that make up this peptide are natural L-chiral configurations. To establish a phenotypic basis for the skin-protective activity of CP161(purity≥99%), we first evaluated its cytotoxicity profile in human dermal fibroblasts (HDFs). Cells were exposed to a wide concentration range of CP161 (0.031–1 mg/mL) for either 24 or 48 h, and cell viability was quantified by the CCK-8 assay. CP161 did not produce any detectable reduction in cell viability across the entire concentration range at either time point, with viability values consistently maintained above 90% relative to untreated controls (**Figures 1B, C**). These results indicate that CP161 is well tolerated by HDFs and define a broad, non-cytotoxic concentration window suitable for subsequent functional characterization.

We next examined whether CP161 could mitigate UVA-induced cellular senescence in HDFs using SA-β-gal staining. UVA irradiation markedly increased the proportion of SA-β-gal-positive cells compared with the untreated control, indicating successful induction of senescence-associated alterations (**Figures 1D, E**). Treatment with CP161 at 10, 60, and 100 μg/mL significantly reduced the percentage of SA-β-gal-positive cells, supporting that CP161 alleviates UVA-induced senescence in HDFs.

Because oxidative stress is a major driver of UVA-induced fibroblast dysfunction, intracellular ROS levels were measured by flow cytometry using the DCFH-DA probe, a widely used method for quantifying oxidative stress responses in cultured cells (Eruslanov and Kusmartsev, 2010). The magnitude of ROS induction observed in our UV model (approximately 30% increase in DCFH-DA mean fluorescence intensity) is consistent with previously reported values for moderate cumulative UVA dosing in primary human dermal fibroblasts (Valencia and Kochevar, 2008; Wang et al., 2019). CP161 co-treatment significantly reduced ROS levels at 10, 60, and 100 μg/mL (**Figure 1F**), indicating that CP161 effectively attenuates UVA-induced intracellular oxidative stress. To further determine whether CP161 also possesses free radical-scavenging activity, a DPPH assay was performed. CP161 displayed a concentration-dependent increase in DPPH radical scavenging activity from 10 to 100 μg/mL (**Figure 1G**), demonstrating that CP161 possesses direct antioxidant capacity in addition to its cellular protective effects.

### 3.2 CP161 promotes extracellular matrix-related gene expression while suppresses MMP1 and p21 protein levels in UVA-treated HDFs

**Figure 2:**
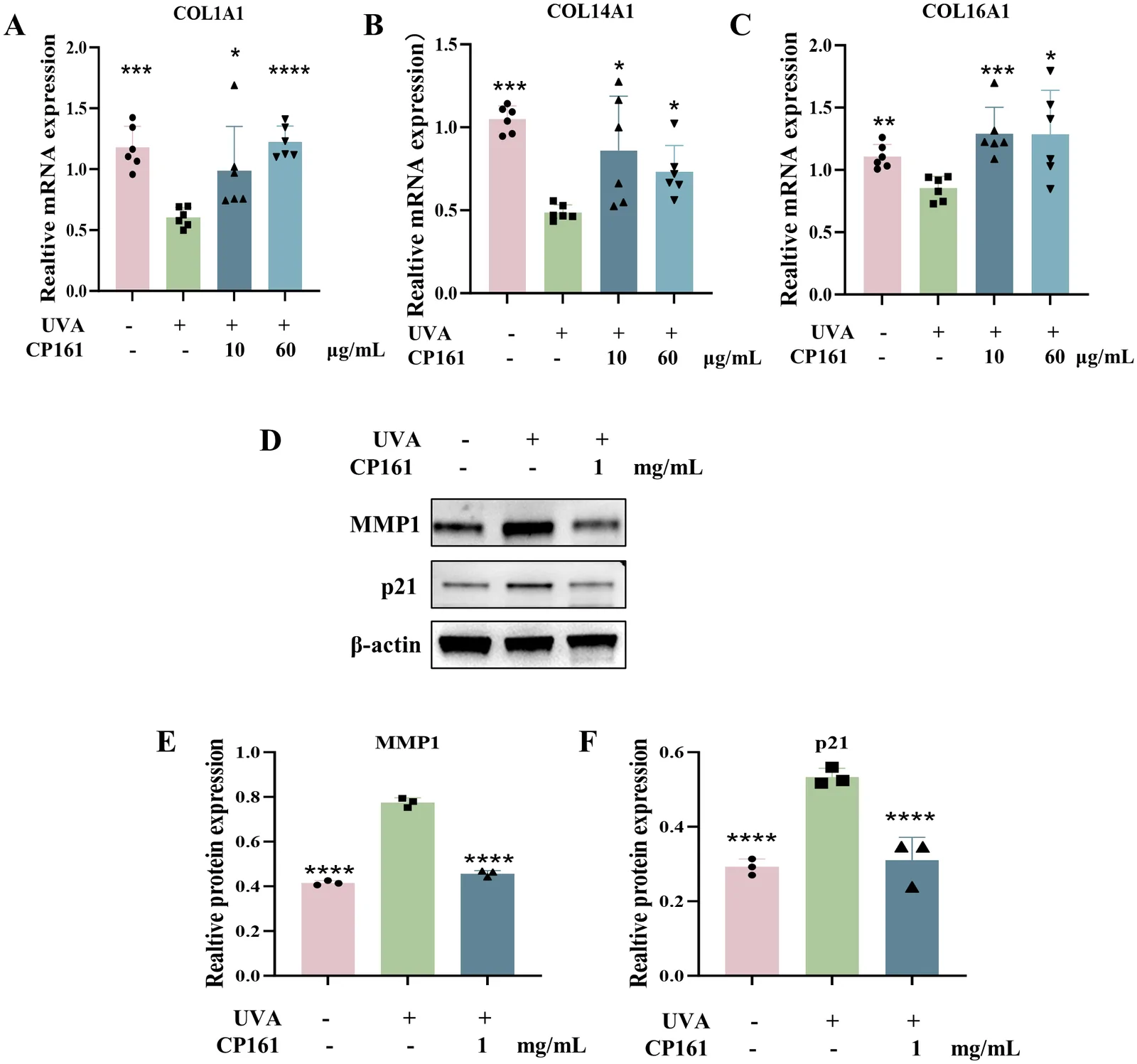
CP161 regulates extracellular matrix-related gene expression. **(A–C)** Relative mRNA expression of *COL1A1* **(A)**, *COL14A1* **(B)**, and *COL16A1* **(C)** in HDFs exposed to UVA irradiation in the presence or absence of CP161 (10 and 60 μg/mL), as determined by qPCR. GAPDH was used as the reference gene. UVA-treated only cells served as the control. Data are presented as mean ± SD (n = 6). *P < 0.05, **P < 0.01, ***P < 0.001, and ****P < 0.0001 versus the UVA-only group. **(D)** Representative western blot images of MMP1 and p21 protein levels in HDFs exposed to UVA irradiation in the presence or absence of CP161 (1 mg/mL). β-actin was used as the loading control. **(E, F)** Quantitative analysis of MMP1 **(E)** and p21 **(F)** protein levels corresponding to **(D)**, normalized to β-actin. Data are presented as mean ± SD (n = 3). ****P < 0.0001 versus the UVA-only group.

Building on the observation that CP161 is biocompatible with HDFs and reduces UVA-induced senescence and oxidative stress, we next examined whether CP161 could further modulate functional outputs that are tightly linked to fibroblast performance, in particular ECM biosynthesis and senescence -associated protein responses. Type I, XIV, and XVI collagens are major fibrillar and fibril-associated components that collectively maintain dermal architecture and mechanical integrity, and their downregulation is a hallmark of UVA-induced ECM dysfunction (Ricard-Blum, 2011; Bonnans et al., 2014). To test whether CP161 could counteract UVA-induced suppression of collagen-related transcripts, the mRNA expression levels of *COL1A1*, *COL14A1*, and *COL16A1* were examined by qPCR. UVA irradiation markedly reduced the expression of all three genes compared with the untreated control (**Figures 2A–C**). Co-treatment with CP161 at 10 and 60 μg/mL significantly restored the expression of *COL1A1*, *COL14A1*, and *COL16A1* relative to the UVA-only group. These results indicate that CP161 promotes collagen-related gene expression in UVA-treated HDFs, suggesting a positive role of CP161 in supporting ECM-related transcriptional programs.

We next examined whether CP161 could also influence senescence-associated proteins that are upregulated under UVA irradiation. MMP1 is a major collagen-degrading enzyme (Pittayapruek et al., 2016), while p21 is a well-established marker of stress- and senescence-associated cell-cycle arrest (Campisi and d'Adda di Fagagna, 2007). Both proteins are commonly upregulated in UVA-injured fibroblasts and contribute to ECM disruption and impaired regenerative capacity. As shown in **Figure 2D**, UVA exposure markedly increased the protein levels of MMP1 and p21 in HDFs, while CP161 treatment substantially attenuated this upregulation. Quantitative analysis further confirmed that CP161 significantly reduced UVA-induced MMP1 (**Figure 2E**) and p21 (**Figure 2F**) protein expression compared with the UVA-only group.

To clarify the concentration-dependent regulatory effect of CP161 on senescence-related proteins and exclude non-specific interference at the high dose of 1 mg/mL, UVA-irradiated human dermal fibroblasts were treated with serial concentrations of CP161 (0, 10, 60, 100, 500,1000 μg/mL), followed by western blot detection of MMP1 and p21. As shown in **Supplementary Figure 1**, the UVA-triggered upregulation of MMP1 and p21 was gradually attenuated with increasing CP161 dosage. This graded response confirmed that the downregulation of MMP1 and p21 arises from specific molecular targeting of CP161, rather than false-positive signals caused by non-specific membrane perturbation or osmotic stress at high concentrations. Given that steady-state protein levels are subject to additional post-transcriptional and post-translational regulation (Liu et al., 2016), at 10 μg/mL, no significant suppression of p21 and MMP1 was observed. However, lower concentration (10μg/mL) may still be sufficient to modulate upstream or membrane-proximal events that regulate collagen gene transcription — a common phenomenon in peptide pharmacology where phenotypic and biochemical readouts exhibit distinct concentration thresholds.

### 3.3 Quantitative proteomics reveals that CP161 modulates extracellular matrix organization and matrix-associated signaling networks in HDFs

To gain a global view of the molecular networks regulated by CP161 in HDFs, quantitative proteomic profiling was performed on three groups: untreated control (BC), UVA-challenged HDFs (NC), and UVA-challenged HDFs treated with CP161 (CP161).

**Figure 3:**
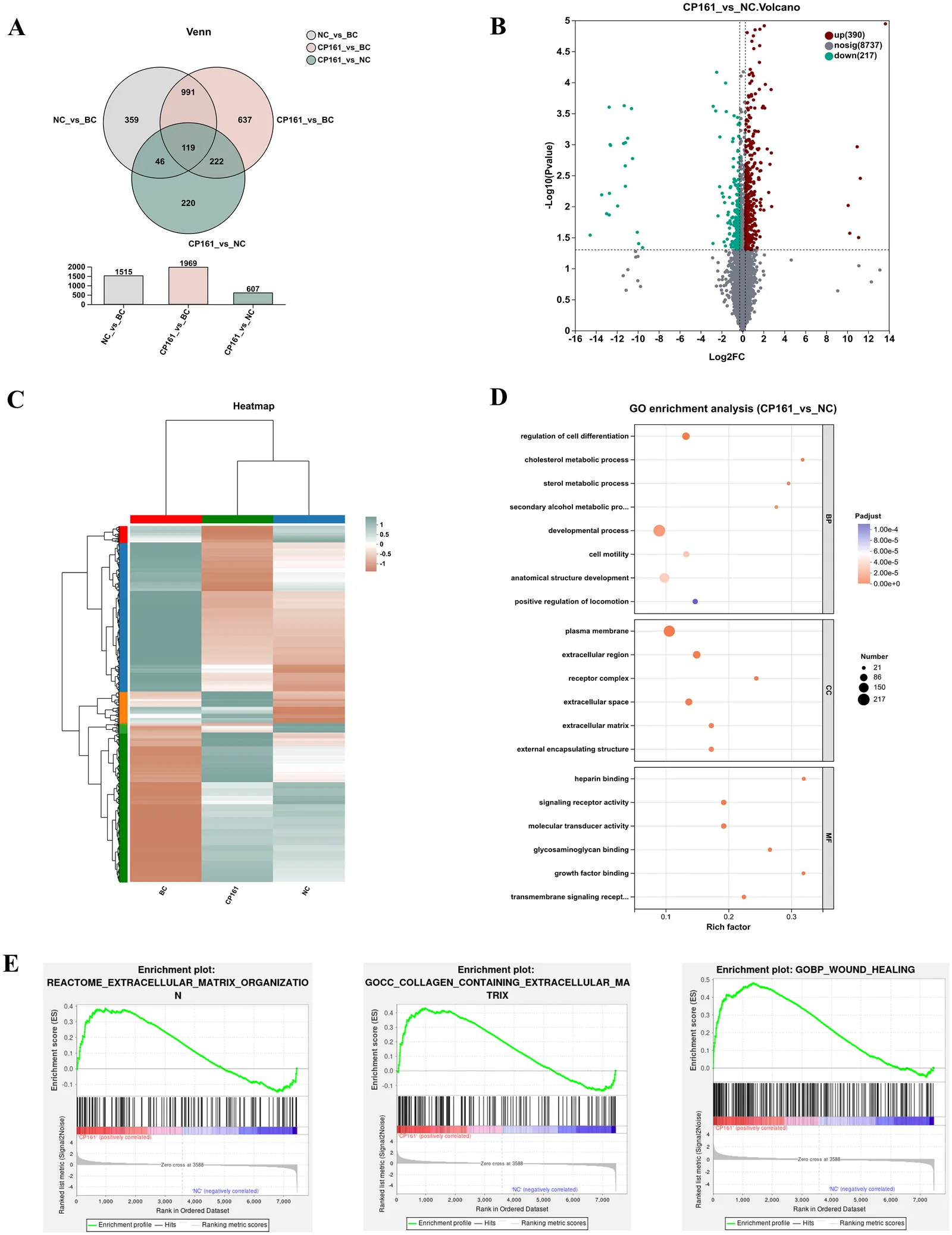
Quantitative proteomic analysis of CP161-treated HDFs. **(A)** Venn diagram showing the overlap of DEPs among the NC_vs_BC, CP161_vs_BC, and CP161_vs_NC comparisons. The bar chart below summarizes the total number of DEPs identified in each comparison. BC, untreated control; NC, UVA-challenged HDFs; CP161, UVA-challenged HDFs treated with CP161. **(B)** Volcano plot of DEPs in the CP161_vs_NC comparison; n = 3 biological replicates per group. Significance was assessed by two-tailed Student’s t-test, and the y-axis displays unadjusted p-values. **(C)** Heatmap of differentially expressed proteins among BC, CP161, and NC groups. **(D)** GO enrichment analysis of DEPs in the CP161_vs_NC comparison. **(E)** GSEA plots showing significant enrichment of the gene sets in CP161-treated HDFs.

Pairwise comparisons among the three groups identified extensive proteomic alterations. Venn analysis revealed substantial overlap as well as group-specific differentially expressed proteins (DEPs) among the NC_vs_BC, CP161_vs_BC, and CP161_vs_NC comparisons (**Figure 3A**), indicating that CP161 treatment markedly remodeled the proteomic landscape of UVA-treated HDFs. Volcano plot analysis of the CP161_vs_NC comparison further showed a clear distribution of significantly upregulated and downregulated proteins (**Figure 3B**), confirming that CP161 induced robust proteomic changes. Heatmap of DEPs across the three groups demonstrated that the proteomic profile of CP161-treated HDFs was clearly distinguishable from that of the NC group (**Figure 3C**).

To define the biological processes and molecular functions associated with CP161-regulated proteins, Gene Ontology (GO) enrichment analysis was performed (**Figure 3D**). In the biological process category, CP161-regulated proteins were significantly enriched in terms closely related to cell function and tissue homeostasis, including regulation of cell differentiation, developmental process, cell motility. In the cellular component category, the enriched terms were dominated by ECM-related compartments, including extracellular region, extracellular space, extracellular matrix, and external encapsulating structure, indicating that CP161-responsive proteins are predominantly localized to the ECM and its surrounding microenvironment.

We next examine these findings at the pathway level using gene set enrichment analysis (GSEA). As shown in **Figure 3E**, CP161 treatment significantly enriched the REACTOME EXTRACELLULAR MATRIX ORGANIZATION, GOCC COLLAGEN CONTAINING EXTRACELLULAR MATRIX, and GOBP WOUND HEALING gene sets, with positive enrichment scores indicating coordinated upregulation of ECM organization, collagen-containing matrix, and wound healing-related programs in CP161-treated HDFs. These pathway-level results are highly consistent with the collagen-related transcriptional restoration and senescence-attenuating effects observed in **Figures 1** and **2**, providing an unbiased confirmation that ECM homeostasis represents a core biological program regulated by CP161 in HDFs.

### 3.4 CP161 directly engages MAP2K1 as a molecular target underlying its ECM-supportive effect in HDFs

The proteomic analyses in **Figure 3** indicated that CP161 converges on ECM organization and matrix-associated signaling networks in HDFs, prompting us to identify the specific molecular node through which CP161 exerts its action. To identify direct molecular targets of CP161, we performed CETSA as an unbiased proteome-wide thermal stability profiling experiment in HDF lysates (Martinez Molina et al., 2013). Melting curves were fitted for all quantified proteins across an 8-point thermal gradient (37–72 °C) using non-linear regression in the TPP R package. The complete list of candidate direct targets and their ΔTm values is provided in **Supplementary Table 2**. Although several candidates exhibited higher ΔTm values (**Supplementary Table 2**), MAP2K1 was selected as the primary focus of mechanistic validation not on the basis of thermal shift magnitude alone, but due to its well-documented, central regulatory role in ECM remodeling and UV-responsive dermal signaling, thereby offering a mechanistically tractable and biologically coherent link to the ECM-centric phenotypes observed in our proteomic and functional assays. Other candidate targets identified by CETSA may be validated in follow-up independent studies.

**Figure 4:**
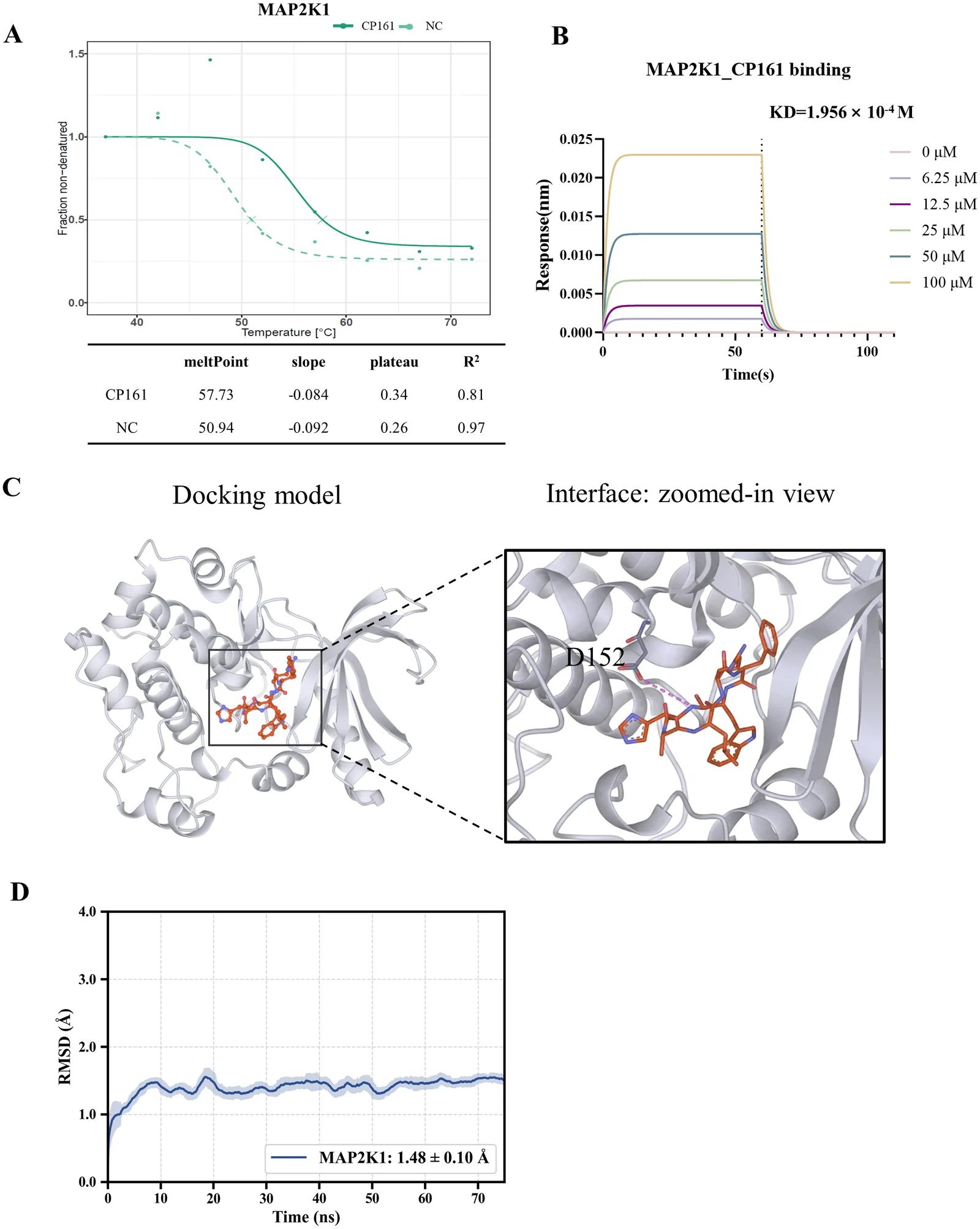
CP161 directly engages MAP2K1 as a molecular target in HDFs. **(A)** CETSA analysis of MAP2K1 in HDFs treated with CP161 or vehicle (NC). The fraction of non-denatured MAP2K1 was plotted against temperature, and curves were fitted to determine the melting temperature (T_m), slope, plateau, and R² values, as summarized in the table below. **(B)** BLI analysis of the direct interaction between CP161 and recombinant MAP2K1. **(C)** Molecular docking model of the CP161–MAP2K1 complex. The overall docked structure is shown on the left, with MAP2K1 (PDB ID:4AN3) displayed as a gray cartoon and CP161 shown in orange. The zoomed-in view on the right highlights the binding interface, including the contact region around residue Asp152. **(D)** Root-mean-square deviation (RMSD) profile of the CP161–MAP2K1 complex during a 50 ns molecular dynamics simulation. The RMSD values stabilized at 1.61 ± 0.12 Å.

MAP2K1 exhibited a significant rightward shift in its melting curve upon CP161 treatment compared with the untreated control (**Figure 4A**). Quantitative fitting showed that the Tm of MAP2K1 increased from 50.94 °C in the control to 57.73 °C in the CP161 group. Curve-fitting parameters further supported the robustness of this stabilization effect, with comparable slopes (-0.084 vs. -0.092), well-resolved plateau values (0.34 vs. 0.26), and acceptable goodness-of-fit values (R^2^ = 0.81 and 0.97 for CP161 and NC, respectively). A thermal shift of this magnitude is well above the threshold typically considered indicative of bona fide ligand–protein engagement in CETSA analyses (Jafari et al., 2014; Franken et al., 2015), providing the first line of evidence that CP161 directly binds MAP2K1 in a cellular environment.

To independently validate this direct interaction, BLI was performed using recombinant MAP2K1. As shown in **Figure 4B**, CP161 exhibited a clear concentration-dependent binding response to immobilized MAP2K1 across the tested range (0–100 μM). Steady-state analysis of the equilibrium response across the concentration gradient yielded an equilibrium dissociation constant (KD) of 1.956 × 10^−4^M for the CP161–MAP2K1 interaction, an affinity that is consistent with that typically observed for cyclic peptide–protein interactions and compatible with the cellular concentrations at which CP161 produced phenotypic effects in UVA-injured HDFs. The equilibrium response was averaged from 50 s to 55 s of the 60 s association phase. Maximum response Rmax = 0.0896 nm, Rmax fitting error = 0.0161 nm. Together, the convergent CETSA-based cellular thermal stabilization and BLI-based biochemical binding evidence establish MAP2K1 as a direct molecular binding partner of CP161.

The CP161–MAP2K1 interaction was then characterized at the structural level by molecular docking. As shown in **Figure 4C**, CP161 was predicted to dock into a defined pocket of MAP2K1, and the zoomed-in view revealed close contacts between the cyclic peptide and key residues at the binding interface, including the region around Asp152, with a calculated binding energy of −4.847 kcal/mol. The cyclic backbone of CP161 enabled a compact and well-fitted engagement with the MAP2K1 binding cavity.

A 75 ns MD simulation was then used to assess the stability of the predicted CP161–MAP2K1 complex, with RMSD monitored throughout the trajectory. The RMSD equilibrated rapidly and remained at 1.48 ± 0.10 Å (**Figure 4D**), a value that is well within the range typically considered indicative of a stably bound and conformationally well-defined ligand–protein complex, with the narrow standard deviation further supporting the absence of large-scale conformational drift or partial dissociation events over the simulation timescale. The MM-PBSA-derived binding free energy (ΔG) was −39.82 ± 15.26 kcal/mol, also suggesting substantial thermodynamic stability of the complex. **Supplementary Figure 3A** presents the 2D interaction diagram of CP161 bound to MAP2K1 generated via PoseView to visualize the hydrogen bonding networks and hydrophobic interactions within the binding pockets.

### 3.5 CP161 suppresses UVA-induced activation of the MAPK signaling cascade in HDFs

We next examined whether CP161 binding translates into functional inhibition of the downstream MAPK signaling cascade. The MEK1/2-ERK1/2 axis is a canonical MAPK signaling module that is rapidly activated under UVA irradiation and plays a central role in driving ECM-degrading and senescence-associated transcriptional programs in dermal fibroblasts (Caunt et al., 2015; Shin et al., 2023). We therefore evaluated the protein levels and activation status of MEK1/2 and ERK1/2 in HDFs.

**Figure 5:**
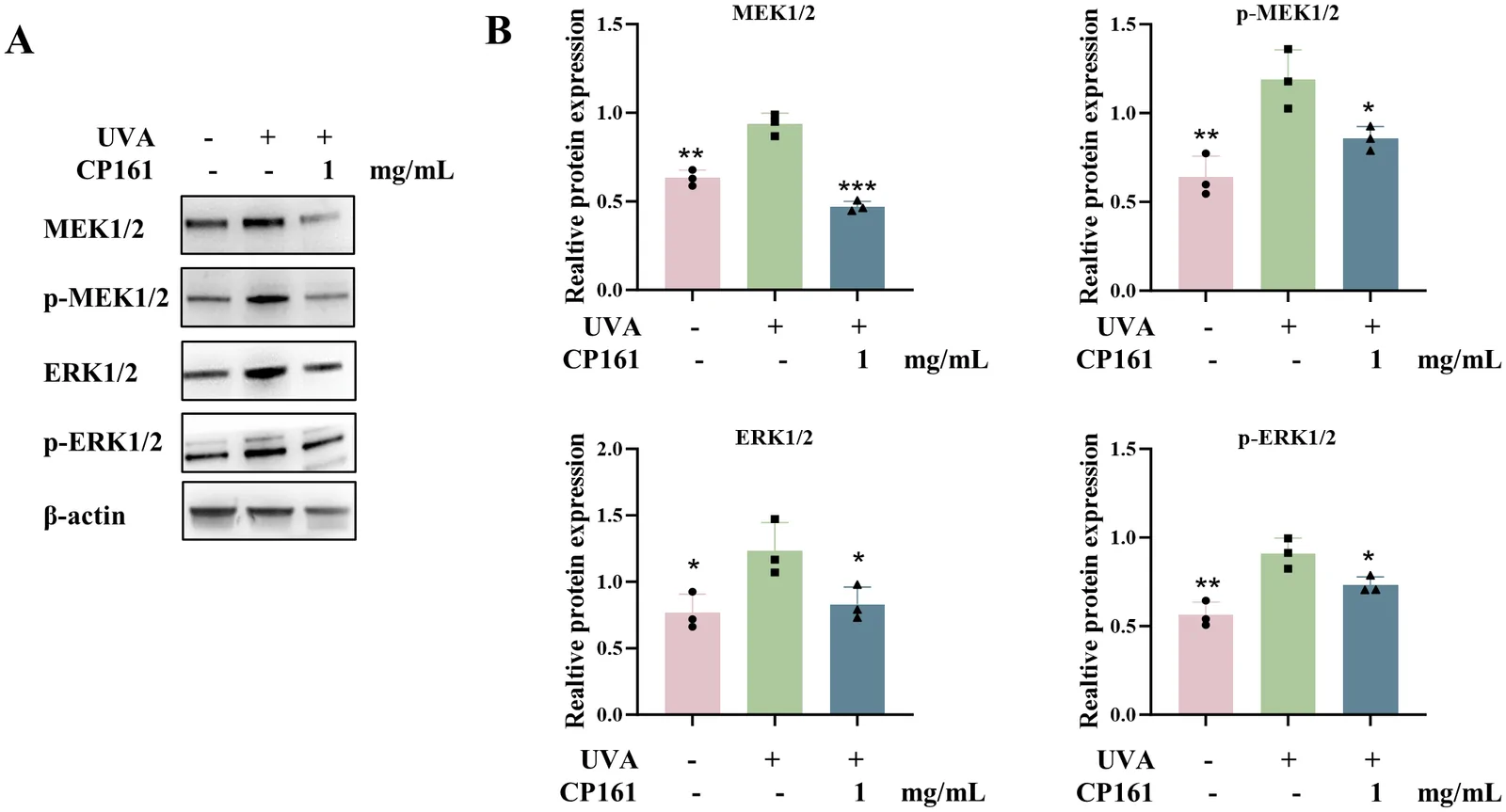
CP161 suppresses UVA-induced activation of the MAPK signaling cascade in HDFs. **(A)** Representative western blot images of MEK1/2, p-MEK1/2, ERK1/2, and p-ERK1/2 protein levels in HDFs exposed to UVA irradiation in the presence or absence of CP161 (1 mg/mL). β-actin was used as the loading control. **(B)** Quantitative analysis of MEK1/2, p-MEK1/2, ERK1/2, and p-ERK1/2 protein levels, normalized to β-actin. *P < 0.05, **P < 0.01, and ***P < 0.001 versus the UVA-only group, n=3.

As shown in **Figure 5A**, UVA irradiation markedly increased the protein levels of MEK1/2, p-MEK1/2, ERK1/2, and p-ERK1/2 in HDFs compared with the untreated control, confirming successful activation of the MEK1/2-ERK1/2 signaling cascade under UVA stress. CP161 treatment at 1 mg/mL substantially attenuated this UVA-induced upregulation across all four protein readouts, indicating that CP161 effectively suppresses both the total expression and the activated forms of the MEK1/2-ERK1/2 axis. Quantitative analysis further confirmed these observations (**Figure 5B**). CP161 treatment significantly reduced UVA-induced MEK1/2 and p-MEK1/2 protein levels relative to the UVA-only group. Consistent changes were observed for the downstream effector ERK1/2 and its phosphorylated form p-ERK1/2, both of which were significantly decreased by CP161 treatment compared with the UVA-only group.

To validate the concentration dependence and specificity of CP161-mediated p-MEK/p-ERK suppression, a dose-response western blot experiment was performed using CP161 at 10, 60, 100, 500, and 1000 μg/mL in UVA-irradiated primary human dermal fibroblasts. CP161 produced a clear suppression of MEK1/2 and ERK1/2 phosphorylation, with significant inhibition observed at 60–500 μg/mL (**Supplementary Figure 2**). Together, these results demonstrate that CP161 effectively suppresses UVA-induced activation of the MEK1/2-ERK1/2 signaling cascade in HDFs, providing functional evidence that CP161 binding to MAP2K1 translates into measurable inhibition of downstream MAPK signaling.

### 3.6 CP161 suppresses acetylcholine release and PTZ-induced hyperlocomotion

**Figure 6:**
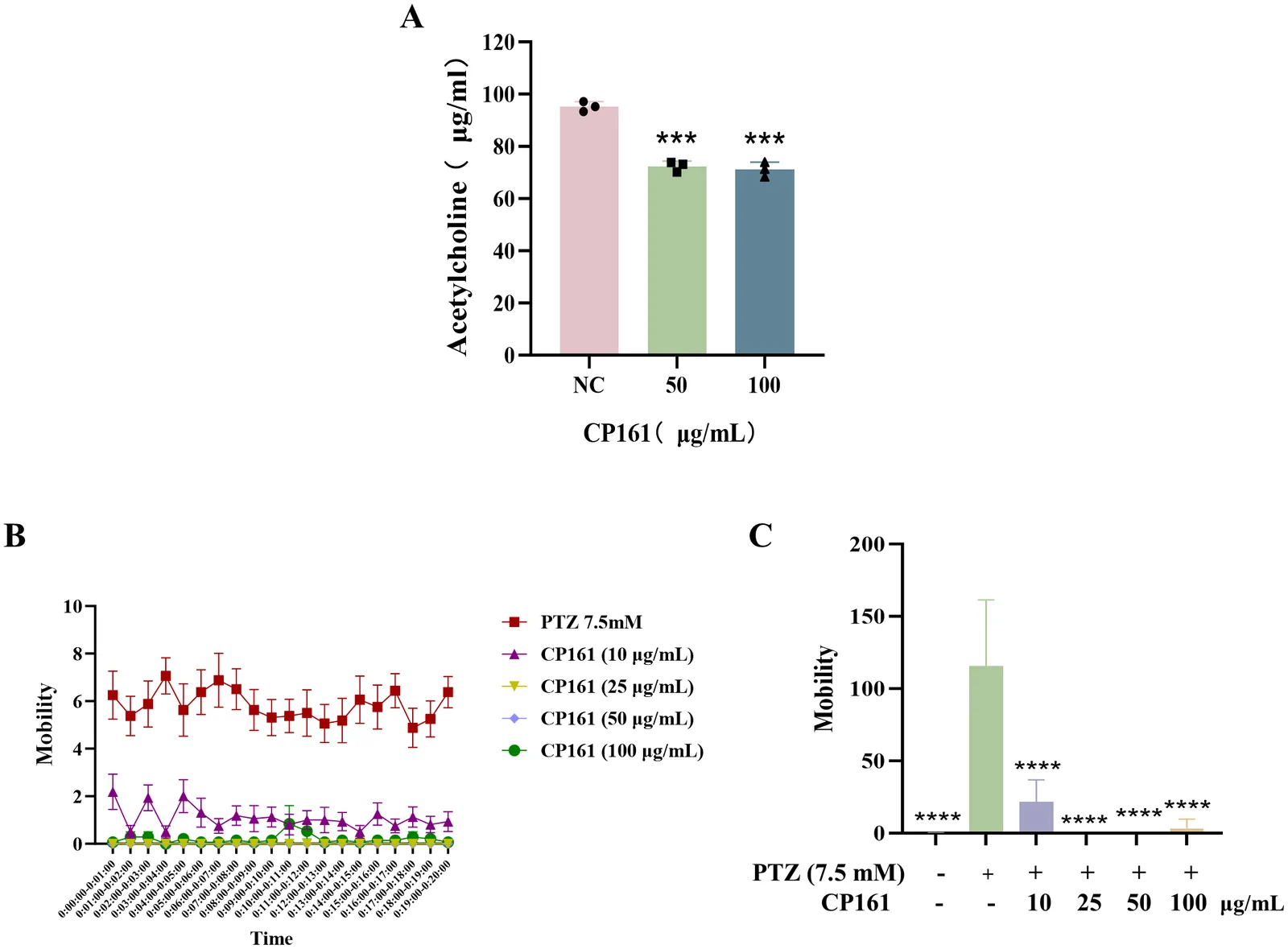
CP161 suppresses acetylcholine release and PTZ-induced hyperlocomotion. **(A)** Quantification of ACh release in hippocampal neuronal cells treated with CP161 at 50 and 100 μg/mL for 24 h. Data are presented as mean ± SD (n = 3). ***P < 0.001 versus the untreated control (NC) group. **(B)** Time-course locomotor activity of 5 dpf zebrafish larvae exposed to 7.5 mM pentylenetetrazole (PTZ) with or without CP161 treatment at 10, 25, 50, and 100 μg/mL. **(C)** Quantification of total zebrafish mobility shown in **(B)**. Data are presented as mean ± SD (n = 24). ****P < 0.0001 versus the PTZ-only group.

Beyond its skin-protective activity, preliminary observations suggested that CP161 might also exert modulatory effects on the Ach release. To investigate this possibility, we first examined whether CP161 alters cholinergic neurotransmitter release in cultured neurons. ACh levels in the supernatant of cholinergic neuronal cultures were quantified following treatment with CP161 at 50 or 100 μg/mL. Compared with the untreated control, CP161 produced a significant reduction in extracellular ACh concentration at both doses (P < 0.001), with the magnitude of suppression being comparable between the two concentrations and reaching approximately 25–30% relative to the control group (**Figure 6A**). These data indicate that CP161 dampens evoked ACh release from cholinergic neurons.

To determine whether this *in vitro* inhibition of cholinergic transmission translates into a measurable phenotype *in vivo*, we employed a pentylenetetrazole (PTZ)-induced zebrafish larval model, an established system for evaluating compounds that modulate neuronal hyperexcitability and neuromuscular activity (Baraban et al., 2005). Zebrafish larvae were exposed to 7.5 mM PTZ in the presence or absence of CP161 at 10, 25, 50, or 100 μg/mL, and locomotor activity was continuously recorded over the assay period. PTZ exposure alone elicited a sustained, high-amplitude increase in larval motility throughout the recording window, consistent with robust seizure-like neuromuscular hyperactivity (**Figure 6B**, red trace). Co-treatment with CP161 produced a dose-dependent attenuation of this response: at the lowest concentration tested (10 μg/mL), motility was substantially reduced relative to PTZ alone, while at concentrations of 25 μg/mL and above, larval movement was suppressed to near-baseline levels and remained essentially flat across the entire recording period (**Figure 6B**). Quantitative analysis of cumulative motility confirmed that PTZ alone produced a marked elevation in larval activity, which was significantly suppressed by CP161 at all concentrations tested (P < 0.0001 for all four CP161 doses versus the PTZ-only group; **Figure 6C**). The 10 μg/mL group retained a low but detectable level of residual motility, whereas the 25, 50, and 100 μg/mL groups reduced motility to levels indistinguishable from non-PTZ controls.

### 3.7 CP161 directly binds Munc18a and forms a stable interaction interface

The suppression of evoked ACh release and PTZ-induced hyperactivity by CP161 prompted us to investigate whether the peptide directly engages a regulator of synaptic vesicle exocytosis. Munc18a (encoded by *STXBP1*) is the principal Sec1/Munc18 family protein in neurons and an obligatory regulator of SNARE complex assembly and calcium-triggered neurotransmitter release, making it a leading candidate target for compounds that modulate evoked transmitter output.

**Figure 7:**
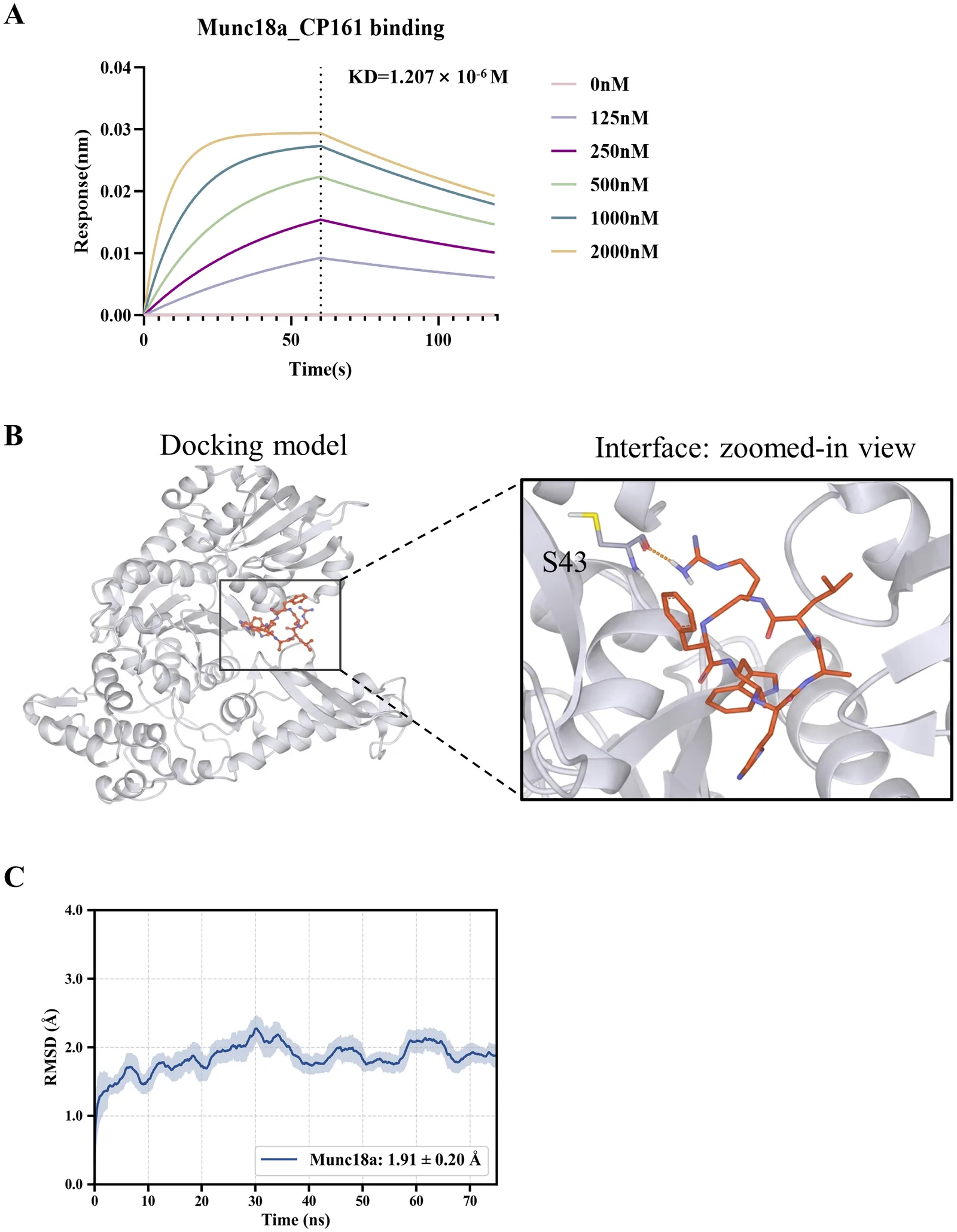
CP161 directly binds Munc18a. **(A)** BLI analysis of the direct interaction between CP161 and recombinant Munc18a. **(B)** Molecular docking model of the CP161–Munc18a complex. The overall docked structure is shown on the left, with Munc18a (PDB ID: 3C98) displayed as a gray cartoon and CP161 shown in orange. The zoomed-in view on the right highlights the binding interface, including the contact region around residue Ser43. **(C)** RMSD profile of the CP161–Munc18a complex during a 50 ns molecular dynamics simulation.

To test direct binding between CP161 and Munc18a, we performed BLI using purified recombinant His-Munc18a immobilized on the biosensor and CP161 as the analyte across a concentration gradient from 0 to 2000 nM. CP161 produced concentration-dependent association and dissociation curves with well-defined steady-state plateaus, and global kinetic fitting yielded a KD of 1.207 × 10^-6^ M (**Figure 7A**). The equilibrium response was averaged from 50 s to 55 s of the 60 s association phase. Maximum response Rmax = 0.0312 nm, Rmax fitting error = 0.0003 nm. This sub-micromolar affinity is consistent with a specific, biologically meaningful interaction between CP161 and Munc18a and confirms that the peptide engages this synaptic regulator directly rather than acting through an indirect upstream mechanism.

To characterize the structural basis of this interaction, we performed molecular docking of CP161 onto the published structure of Munc18a. The docking site selection was based on the published crystal structure of the Munc18a-Syntaxin 1a complex (PDB ID: 3C98). Structural analysis has revealed that the H3 domain of Syntaxin 1a and the domain 3a of Munc18a form a critical protein-protein interaction interface, which is essential for regulating SNARE complex assembly. Previous studies have shown that point mutation of the C-terminal I233 residue within the Syntaxin 1a H3 domain (I233A) significantly reduces the binding affinity between Munc18a and Syntaxin 1a (Burkhardt et al., 2008). Based on this evidence, the docking grid was defined to encompass this core interaction interface, covering key Munc18a residues including S43 and C45, which correspond to the binding pocket accommodating the I233 residue of Syntaxin 1a, thereby ensuring the structural rationale of the docking mode.

The lowest-energy docking poses placed CP161 within a defined surface pocket of Munc18a, with the cyclic peptide adopting a compact conformation that engaged the binding cleft through multiple contacts (**Figures 7B**, left). A zoomed-in view of the binding interface revealed that CP161 lies in close proximity to Ser43 of Munc18a, a residue located within a functionally important regulatory region of the protein, with a calculated binding energy of −7.523 kcal/mol. (**Figures 7B**, right). The docking pose indicated that CP161 occupies a surface groove on Munc18a that is positioned to influence the conformational dynamics of the SNARE-binding interface, providing a structural rationale for the functional inhibition observed in cellular and zebrafish assays.

To assess whether the predicted CP161–Munc18a complex is structurally stable under physiological conditions, we performed a 75 ns all-atom MD simulation in explicit solvent. The RMSD of the Munc18a backbone relative to its starting conformation rose rapidly during the initial equilibration phase and then plateaued at approximately 2 Å, with a mean value of 1.91 ± 0.20 Å maintained throughout the remainder of the trajectory (**Figure 7C**). The MM-PBSA-derived binding free energy (ΔG) was −36.17 ± 12.11 kcal/mol, demonstrating substantial thermodynamic stability. **Supplementary Figure 3B** illustrates the 2D docking interaction between CP161 and Munc18a generated via PoseView to visualize the hydrogen bonding networks and hydrophobic interactions within the binding pockets. These results together indicate that the CP161-bound Munc18a complex is conformationally stable across the simulation window and that the binding pose identified by docking represents a physically realistic engagement mode rather than a transient interaction.

## 4 Discussion

Cutaneous senescence arises from a self-reinforcing molecular circuit in which UVA-induced oxidative stress drives sustained MAPK activation, transcription of matrix-degrading enzymes, transcriptional suppression of fibrillar collagens, and progressive accumulation of senescent fibroblasts that further amplify dermal deterioration. Effective intervention in this circuit requires engagement of a node that is simultaneously rate-limiting for the cascade and amenable to modulating cutaneous homeostasis. The present study identifies the cyclic hexapeptide CP161 as a peptide modulator that addresses this requirement by directly engaging MAP2K1, attenuating UVA-induced ERK1/2 phosphorylation, and coordinately restoring the major phenotypic features of UVA-injured dermal fibroblasts—including ECM gene expression, ROS accumulation, and stress-induced skin senescence.

Quantitative proteomics provided a system-level view and revealed that CP161 reshapes the proteomic landscape of UVA-treated HDFs, with GO enrichment and GSEA converging on extracellular matrix organization, collagen-containing extracellular matrix, and wound healing. These pathway-level findings are highly consistent with the qPCR and western blot results, providing orthogonal evidence that ECM homeostasis represents a core biological program modulated by CP161.

The convergence of CETSA, BLI, molecular docking, and molecular dynamics simulation on MAP2K1 as the direct binding partner of CP161 places this kinase at the mechanistic center of the peptide’s skin-protective activity. MAP2K1 occupies a uniquely strategic position in UVA-responsive signaling: as the key kinase linking downstream ERK1/2 effectors, it is rate-limiting for the AP-1 induced transcriptional program that simultaneously governs MMP-driven ECM degradation, procollagen suppression, and the establishment of stress-induced skin senescence. Engagement at this single node therefore provides a mechanistically explanation for the breadth of phenotypic effects produced by CP161 treatment. The coordinated restoration of *COL1A1*, *COL14A1*, and *COL16A1* expression alongside suppression of MMP1 indicates that CP161 reaches a node sufficiently upstream to reset matrix homeostasis rather than acting on individual ECM components in isolation, an effect that is precisely what would be predicted from attenuation of MEK1/2-ERK1/2 signaling. The reduction in p21 expression and SA-β-gal accumulation under UVA stress further indicates that CP161 prevents the establishment of stress-induced skin senescence, a property that is mechanistically consistent with engagement of a signaling node upstream of the senescence-establishing transcriptional program. This is particularly significant given that prevention of senescence onset, rather than clearance of already-damaged cells, represents the more attractive therapeutic strategy for chronically sun-exposed skin in which UVA exposure is recurrent and cumulative.

The concurrent increase in total and phosphorylated MEK1/2 and ERK1/2 observed in our study may reflect the relatively higher cumulative UVA dose and repeated irradiation regimen used to model photodamage, compared with the lower-dose or shorter-duration protocols employed in some previous studies (Seo et al., 2018; Xie et al., 2021), suggesting that UV-induced changes in MAPK abundance and activation are dose- and regimen-dependent. Specifically, sustained UV exposure activates the AP-1 (c-Jun/c-Fos) transcriptional program, which upregulates MAPK cascade components and expands the total MEK/ERK protein pool (Angel et al., 2001; Silvers et al., 2003). In parallel, chronic oxidative stress impairs proteasomal function in primary fibroblasts, slowing the ubiquitin-proteasome-mediated turnover of MAPK proteins and further contributing to their accumulation (Beach and Egelhoff, 2009). Together, these transcriptional and degradative mechanisms account for the concordant increase in total and phosphorylated kinase levels in our system. Importantly, the CP161-mediated inhibition of MEK/ERK phosphorylation was independently confirmed by the newly added dose-response western blot (**Supplementary Figure 2**), supporting the specificity of its effect under chronic photoaging-relevant conditions.

The moderate BLI-derived affinity of CP161 for MAP2K1 (K_D ≈ 196 μM) may appear inconsistent with the robust phenotypic effects on collagen gene expression observed at 10–60 μg/mL. However, this is a common feature of peptide-class modulators acting at protein–protein interaction interfaces rather than through ATP-competitive binding, which typically function in the high-nanomolar to sub-millimolar affinity range and can still elicit strong cellular responses once sufficient target occupancy is achieved. Moreover, the pronounced signal amplification inherent to the MEK/ERK cascade allows even partial upstream engagement to translate into substantial downstream transcriptional changes, accounting for the potent collagen-promoting activity at low working concentrations.

Beyond its direct effects on UVA-injured fibroblasts, CP161 also exhibits an activity at the neuromuscular interface that is mechanistically distinct from but functionally complementary to its dermal action. CP161 directly binds the synaptic regulator Munc18a, an obligatory upstream regulator of SNARE complex assembly and calcium-triggered neurotransmitter exocytosis, and produces a measurable, dose-dependent suppression of evoked acetylcholine release from cholinergic neurons as well as robust attenuation of PTZ-induced neuromuscular hyperactivity in zebrafish larvae. This activity is mechanistically relevant to a long-recognized but molecularly underexplored aspect of skin pathology: repeated and sustained contraction of the underlying musculature exerts mechanical force on the overlying dermis, and this chronic mechanical loading drives a distinct, biomechanically initiated mode of dermal injury. Sustained mechanotransduction at the dermal fibroblast level perturbs the balance between collagen synthesis and turnover, accelerates fibroblast senescence in a manner that is mechanistically convergent with the UVA-driven oxidative and MAPK-mediated pathway. Skin overlying chronically contracting musculature therefore experiences a dual insult: intrinsic UVA-driven biochemical damage on one hand, and extrinsic mechanical strain–driven cellular and matrix injury on the other. The attenuation of acetylcholine release by CP161 through Munc18a engagement is mechanistically positioned to dampen the upstream neuromuscular drive of this mechanical injury process. Considered together with the MAP2K1-mediated dermal protective effects, the two activities of CP161 therefore converge on functionally complementary axes of dermal injury—biochemical UVA-driven damage on one side, and biomechanical contraction-driven senescence on the other—providing a mechanistically integrated rationale for the multi-faceted skin-protective profile of this peptide. However, we must acknowledge that the experimental models used in this study have certain limitations. Future studies may consider employing neuromuscular junction models to further validate our conclusions.

CETSA in the present study was performed on cellular lysates rather than live intact cells. While lysate-based CETSA enables unbiased proteome-wide profiling of thermal stability shifts across thousands of proteins simultaneously (Martinez Molina et al., 2013), it cannot fully exclude the possibility that some observed interactions occur as artifacts of the cell disruption process. The convergence of lysate CETSA with orthogonal cell-free methods (BLI with purified recombinant MAP2K1, molecular docking, and MD simulation) provides strong complementary evidence for a direct physical interaction independent of the lysate context. Future studies employing intact-cell CETSA or co-immunoprecipitation would further strengthen claims of intracellular target engagement under physiological conditions.

It should be noted that the CETSA screening described above was performed exclusively in HDF lysates; consequently, Munc18a—a synaptic protein predominantly expressed in neuronal tissue and not captured by this fibroblast-based CETSA screen—was identified as a candidate CP161-interacting protein through independent BLI and computational analyses, rather than via the HDF-derived CETSA dataset presented in **Supplementary Table 2**.

Another key limitation is that all findings derive from cultured HDFs and zebrafish larvae; validation in *ex vivo* human skin explants, 3D reconstructed skin equivalents, or *in vivo* photoaging animal models will be essential to translate these mechanisms toward dermatological application.

In summary, the present study identifies the cyclic hexapeptide CP161 as a mechanistically defined modulator of UVA-induced dermal injury that acts through direct engagement of MAP2K1, and engagement of this kinase attenuated UVA-induced ERK1/2 phosphorylation, restored fibrillar collagen expression, suppressed MMP1 and p21 induction, and reduced intracellular ROS in human dermal fibroblasts. A complementary activity at the neuromuscular regulator Munc18a was also identified, suggesting a broader protective potential against contraction-associated mechanical injury to the dermis. Collectively, these findings establish CP161 as a mechanistically resolved cyclic peptide modulator of MAP2K1-dependent pathways and provide a generalizable framework for the in-depth molecular characterization of skin-protective cyclic peptides.

## Data Availability

The datasets presented in this study can be found in online repositories. The names of the repository/repositories and accession number(s) can be found below: https://www.iprox.cn/page/home.html, IPX0017827000.
