# Supplementary material for: Cyclopeptide 161 protects human dermal fibroblasts against UV-induced damage via the MEK1/2–ERK1/2 signaling pathway
Source: Front Physiol. 2026 Sep 9;17:1912562. doi: 10.3389/fphys.2026.1912562 (PMC13597423; doi:10.3389/fphys.2026.1912562)
Supplement: Supplementary file 1 [file DataSheet1.docx]

Table S1. Primer sequences for qRT-PCR.

| **Gene** | **Primer sequence (5’-3’)** |
| --- | --- |
| COL1A1_F | GAGAGCATGACCGATGGATT |
| COL1A1_R | АСТТGCCATCTGACAGGTTGA |
| COL14A1_F | GCTCAAAGAGGAATTGGGAAA |
| COL14A1_R | TTCTTTTCCTGCTAGGGACAT |
| COL16A1_F | CCACCAGAAGACGTGGTATCT |
| COL16A1_R | CAGGACACAAAGTCGCCATC |
| GAPDH-F | TGCACCACCAACTGCTTAGC |
| GAPDH-R | GGCATGGACTGTGGTCATGAG |

Table S2. Candidate targets of CP161 identified by CETSA.

| **Gene names** | **ΔTm** |
| --- | --- |
| RDH14 | 24.29072525 |
| MRPL53 | 17.71963962 |
| MFN1 | 17.55009009 |
| HMGCS1 | 16.9925177 |
| ALG13 | 16.90798449 |
| SEC23B | 16.36097719 |
| NDC80 | 13.80458576 |
| TRMT1L | 13.22217167 |
| GLT8D2 | 13.11546145 |
| SCRN2 | 12.7569626 |
| SMAD2 | 6.827835813 |
| MAP2K1 | 6.791337882 |
| TOMM40 | 6.110817106 |

**Figure S1. Dose-dependent effects of CP161 on MMP1 and p21 protein expression in UV-irradiated primary human dermal fibroblasts.**

(A) Representative western blot images showing MMP1, p21, and β-actin protein levels in fibroblasts treated with vehicle control, UV irradiation alone, or UV irradiation combined with increasing concentrations of CP161 (10, 60, 100, 500, and 1000 μg/mL). β-actin was used as the internal loading control. (B, C) Quantification of relative protein expression levels of p21 (B) and MMP1 (C), normalized to β-actin. Data are presented as mean ± SD (n = 3). Statistical comparisons were made against the UVA-only group. ns, not significant; *P < 0.05; **P < 0.01; ***P < 0.001; ****P < 0.0001.

**Figure S2. Dose-dependent inhibition of MEK/ERK pathway activation by CP161 in UV-irradiated primary human dermal fibroblasts.**

(A) Representative western blot images showing p-MEK1/2, total MEK1/2, p-ERK1/2, total ERK1/2, and β-actin protein levels in fibroblasts treated with vehicle control, UV irradiation alone, or UV irradiation combined with increasing concentrations of CP161 (10, 60, 100, 500, and 1000 μg/mL). (B–E) Quantification of relative protein expression levels of total ERK1/2 (B), phosphorylated ERK1/2 (p-ERK1/2) (C), total MEK1/2 (D), and phosphorylated MEK1/2 (p-MEK1/2) (E), all normalized to β-actin. Data are presented as mean ± SD (n = 3). Statistical comparisons were made against the UVA-only group. ns, not significant; *P < 0.05; **P < 0.01; ***P < 0.001; ****P < 0.0001.

**Figure S3. Two-dimensional (2D) interaction diagrams of cyclopeptide-161 with target proteins.**

(A) Binding mode of cyclopeptide-161 in the MAP2K1 complex. (B) Binding mode of cyclopeptide-161 in the Munc18a complex. Black dashed lines represent hydrogen bonds, with numbers indicating the bond lengths (in Å); green solid lines indicate hydrophobic interactions formed between the receptor residues and the ligand.

**Figure S4. Representative amplification plots of real-time qPCR analysis for collagen genes COL1, COL14, and COL16.**

Amplification plots show the fluorescence signal (ΔRn) as a function of PCR cycle number for the target genes COL1, COL14, and COL16 and the reference housekeeping gene GAPDH (as indicated) across all replicates.

**Figure S5. Original uncropped western blot images corresponding to Figure 2D.**

**Figure S6. Original uncropped western blot images corresponding to Figure 5A.**

**Figure S7. Original uncropped western blot images corresponding to Figure S1A.**

**Figure S8. Original uncropped western blot images corresponding to Figure S2A.**
